# Impact of age on plasma vaspin concentration in a group of normal Chinese people

**DOI:** 10.1007/s40618-016-0533-6

**Published:** 2016-09-07

**Authors:** X. Xu, J. Wen, Y. Lu, H. Ji, J. Zhuang, Y. Su, B. Liu, H. Li, Y. Xu

**Affiliations:** Shanghai Tenth People’s Hospital, Tongji University School of Medicine, Shanghai, China

**Keywords:** Adipokines, Vaspin, Ageing, Insulin resistance

## Abstract

**Purpose:**

Visceral adipose tissue-derived serine protease inhibitor (vaspin) is an adipocytokine with insulin-sensitizing effects. Accumulating data implied that vaspin represents a compensatory mechanism but it is unknown how vaspin change during ageing. This study was designed to examine the correlation between plasma vaspin and age in a group of normal Chinese people.

**Methods:**

A total of 191 Chinese volunteers aged 19–80 years were enrolled into four groups based upon age quartiles (19–35, 36–50, 51–65 and 66–80 years). Demographic, anthropometric, metabolic covariates, vaspin and adiponectin were measured. The influence of age on plasma vaspin was analysed using SPSS 13.0.

**Results:**

Vaspin increased with ageing, with mean vaspin levels (ng/mL) of 1.01 ± 2.25, 1.67 ± 2.95, 2.05 ± 3.46 and 2.40 ± 3.06 for those between quartile ages 19–35, 36–50, 51–65 and 66–80 years. When divided into subgroups, vaspin increased with increasing age for both sexes, both insulin resistance and non-insulin resistance subjects and both obese and lean subjects. In univariate analyses, vaspin plasma level positively associated with age (*r* = 0.215, *p* = 0.003), adiponectin, insulin, homoeostasis model of assessment for insulin resistance index and waist–hip ratio in the whole population. The correlation between ageing and increasing vaspin remained significant after multivariate adjustments for factors such as sex, body mass index, waist–hip ratio, indices of glucose metabolism, white blood cell, lipid profile and adiponectin. Stepwise multiple regression analysis showed that age contributed 7.6 % on plasma vaspin level.

**Conclusion:**

Vaspin level increased with ageing, independent of sex, indices of glucose metabolism, lipid profile and other markers of adiposity.

## Introduction

Adipose tissue, previously perceived to be a storage reservoir of fat, is now recognized as an active endocrine organ [1, 2]. Adipose tissue secretes a number of adipocyte-specific cytokines (adipokines), which played important roles in the process of the metabolism, inflammation, cell proliferation and apoptosis [3, 4]. Ageing is correlated with an increase in fat mass and a redistribution of adipose tissue from peripheral to central fat depots [5]. Adipokine dysregulation, as a common ground for insulin resistance, is also paradoxically correlated with lipodystrophy and lipoatrophy with ageing [6]. A recent study, which used centenarians as a model of healthy ageing and longevity, also suggests that adipose tissue excess as well as its ageing would regulate the expression of adipokines [7].

Vaspin is an interesting adipocytokine with insulin-sensitizing effects [8–10]. Accumulating data implied that vaspin represents a compensatory mechanism in the development of obesity and metabolic disorders [8, 11–13]. Up-regulation of vaspin may have a protective effect against insulin resistance [9, 10, 14]. Central vaspin administration leads to reduced food intake and has sustained blood glucose-lowering effects [15]. As basic studies reported, it could suppress the expression of several inflammatory factors such as tumour necrosis factor (TNF)-α, leptin, resistin and adiponectin [10, 16], inhibit TNF-α-induced expression of adhesion molecules in vascular smooth muscle cells and blunt later lymphocyte adhesion [17], inhibit proliferation, chemokinesis and reactive oxygen species production of vascular smooth muscle cells [18, 19], and also protect vascular endothelial cells against free fatty acid-induced apoptosis [20]. Improved insulin resistance, attenuated inflammation and anti-apoptosis suggest that vaspin has a protective function.

Although previous studies suggested there may be some correlation between age and circulating vaspin level, the results were ambiguous and indefinite. Some have shown an age-related increase [13, 21–24], whereas others found no effect [25, 26] or even an age-related decrease [27] in serum vaspin concentrations. The contradiction may due to different experimental design and study population with narrow-spectrum ages. This paper was designed to examine the correlation between plasma vaspin level as a continuous variable and age, in a well-characterized, wide age-ranged, healthy group of Chinese people. We also determined whether any age-related changes in plasma vaspin were explained by metabolic syndrome components, adiposity-related biomarkers such as BMI, waist–hip ratio, adiponectin and insulin.

## Methods

### Study population

The subjects included 191 individuals (106 men and 85 women) who underwent complete medical check-ups at the Physical Checkup Centre at Shanghai Tenth People’s Hospital (China). The subjects included in this study were representative of normal Chinese people because they were patients who had not attended our hospital, who had no apparent disease that required hospital admission, and people were all ages and occupations. People who were diagnosed as diabetes mellitus, hypertension or dyslipidaemia, or receiving any medications that affect blood pressures, body weight, glucose and lipid levels were excluded. Subjects were considered to have hypertension if they had a mean systolic blood pressure (SBP) ≥140 mmHg and/or diastolic blood pressure (DBP) ≥90 mmHg and/or used anti-hypertensive medications. Diabetes was diagnosed according to the WHO criteria. Exclusion criteria for all study participants also include smoking, pregnancy, acute infection, hepatic dysfunction or renal dysfunction, neoplasm, haematologic disorders. Those who consumed at least one cigarette per day were considered smokers. Subjects who had never or stopped smoking during the last 12 months after years of smoking were considered non-smokers. All study participants were given written informed consent, and the study protocol was approved by Shanghai Tenth Hospital’s Ethics Committee.

### Anthropometric and clinical assessments

All study participants underwent a standard clinical examination. A trained interviewer gathered information regarding medical history and current medication use. Height and body mass were recorded using a stadiometer attached to a scale. Weight was obtained with participants wearing light clothing and no shoes. Waist circumference measurements were made using a cloth tape measure at the level of the umbilicus. Hip girth was measured as the horizontal circumference at the broadest part of the lower body, usually at the level of the trochanters. BMI was calculated as weight divided by the square of height. In the present analyses, we assigned individuals to non-obese and obese subgroups based on a modified cut-off value of BMI ≥ 25.0 kg/m^2^ for classification of obesity from an Asia-Pacifica perspective. The index of insulin resistance was assessed using the homoeostasis model of assessment–insulin resistance [28] (HOMA-IR), as HOMA-IR = [fasting insulin (μU/mL) × fasting glucose (mM)]/22.55 [29]. Insulin resistance (IR) was defined as the levels of the HOMA-IR greater than 2.55 [29].

### ELISA assays and biochemical investigations

5 mL whole blood samples is obtained from all of the normal subjects before their medical check-up (more precisely, it was after overnight fast), in tubes containing EDTA for adipokines and biochemical investigations. Blood samples were centrifuged at 1000*g* for 10 min. Plasma specimens were then frozen and stored at −80 °C until analysis. Human total adiponectin (R&D Systems, Minneapolis, USA) and vaspin (Adipogen, Seoul, South Korea) plasma levels were measured with ELISAs which had been reported previously [26]. Fasting insulin concentrations were measured with a commercially available ELISA immunoassay kit (ALPCO Diagnostics, Salem, NH, USA). White blood cell count, high-sensitive C-reactive protein (hsCRP), fasting plasma glucose (FPG), alanine aminotransferase (ALT), blood urea nitrogen (BUN) and lipid profiles including total cholesterol (TC), low-density lipoprotein cholesterol (LDL-C), high-density lipoprotein cholesterol (HDL-C), triglycerides (TG), uric acid (UA) and creatinine (Cr) were measured by colorimetric enzymatic assay systems (Roche MODULAR P-800, Swiss Confederation).

### Statistical analysis

Subjects were divided into four groups based upon age quartile (19–35, 36–50, 51–65 and 66–80 years). Continuous variables were described as mean ± SD. Categorical variables were presented as frequencies. Normal distribution was verified with the Kolmogorov–Smirnov test. All variables were checked for normality and subjected to log10 transformations, if necessary, prior to statistical analyses, but the actual data were presented. Comparisons between groups were made using unpaired *t* tests, ANOVA or a nonparametric Mann–Whitney *U* test when appropriate. Chi-square test or Fisher’s exact test was employed for nonparametric data. Spearman’s rank correlation test and multiple linear regression analyses were used to determine the degree to which the variance in plasma vaspin levels can be explained by single factors. A two-sided probability level of ≤0.05 was taken as significance. All analyses were done with SPSS for Windows 13.0 (SPSS Inc, Chicago, Illinois, USA).

## Results

### Subjects characteristics

The basic characteristics of the participants are shown in Table 1. There were 191 study participants that contained 106 men (55.7 %) and 85 women (44.5 %). Mean ± SD age of the participants was 50 ± 17 years, ranged 19–80 years. Average of vaspin and adiponectin was 1.78 ± 2.99 ng/mL and 11.79 ± 8.91 μg/mL, respectively. The subjects were enrolled into the following four groups based upon the age quartiles: 19–35 (*n* = 48), 36–50 (*n* = 47), 51–65 (*n* = 48) and 66–80 (*n* = 48) years. Gender, lipid profile, hsCRP and insulin showed no difference among different age groups. BMI, waist–hip ratio, FPG, IR prevalence (IR in Table 1), HOMA-IR, both systolic and diastolic blood pressure were increased with age.

**Table 1:** Clinical and biochemical characteristics of the study population

Parameter	Age quartiles				
	19–35 (*n* = 48)	36–50 (*n* = 47)	51–65 (*n* = 48)	66–80 (*n* = 48)	*p* value
Male [*n* (%)]	24 (50 %)	22 (47.8 %)	35 (72.9 %)	25 (52.1 %)	0.332
IR [*n* (%)]	9 (18.8 %)	13 (27.7 %)	14 (29.2 %)	16 (33.3 %)	0.116
BMI (kg/m^2^)	21.24 ± 2.79	24.10 ± 3.36	24.48 ± 2.56	24.22 ± 2.80	<0.001
Waist–hip ratio	0.81 ± 0.65	0.83 ± 0.10	0.87 ± 0.85	0.87 ± 0.10	0.002
SBP (mmHg)	117 ± 12	130 ± 8	127 ± 8	128 ± 9	<0.001
DBP (mmHg)	72 ± 10	82 ± 9	80 ± 9	75 ± 10	<0.001
TC (mmol/L)	4.41 ± 1.02	4.82 ± 1.12	4.67 ± 1.00	4.68 ± 0.83	0.250
HDL-C (mmol/L)	1.26 ± 0.31	1.22 ± 0.31	1.19 ± 0.25	1.13 ± 0.20	0.203
LDL-C (mmol/L)	2.51 ± 0.76	2.78 ± 0.73	2.66 ± 0.69	2.64 ± 0.63	0.300
TG (mmol/L)	1.21 ± 0.69	1.64 ± 0.81	1.50 ± 0.78	1.48 ± 0.66	0.041
FPG (mmol/L)	4.70 ± 0.64	5.32 ± 0.70	5.25 ± 0.74	5.14 ± 0.77	<0.001
WBC (×10^9^/L)	6.07 ± 1.24	5.81 ± 1.34	6.63 ± 1.51	6.41 ± 1.71	0.035
hsCRP (mg/L)	3.73 ± 2.05	6.69 ± 5.57	6.54 ± 5.93	6.59 ± 6.08	0.882
ALT (U/L)	14.82 ± 7.50	21.67 ± 15.80	26.26 ± 18.44	19.60 ± 13.21	0.002
BUN (mmol/L)	6.17 ± 8.32	6.85 ± 10.33	5.77 ± 1.19	6.73 ± 5.62	0.870
Cr (mg/L)	71.54 ± 30.64	76.72 ± 51.97	84.37 ± 47.74	85.22 ± 37.58	0.343
uric acid (mg/L)	312.45 ± 91.77	298.64 ± 108.06	350.99 ± 94.36	369.77 ± 110.61	0.002
Vaspin (ng/mL)	1.01 ± 2.25	1.67 ± 2.95	2.06 ± 3.49	2.40 ± 3.06	0.123
Adiponectin (μg/mL)	8.95 ± 5.76	10.45 ± 11.26	10.985 ± 6.34	16.78 ± 9.34	<0.001
Insulin (uIU/mL)	9.20 ± 5.32	9.47 ± 4.20	9.65 ± 3.86	10.30 ± 3.34	0.625
HOMA-IR	1.90 ± 1.06	2.26 ± 1.096	2.24 ± 0.93	2.35 ± 0.83	0.131

Continuous variables were described as mean ± SD; categorical variables were presented as frequencies

*SBP* systolic blood pressure, *DBP* diastolic blood pressure, *n* number of patients, *BMI* body mass index, *LDL*-*C* low-density lipoprotein cholesterol, *HDL*-*C* high-density lipoprotein cholesterol, *TG* triglycerides, *TC* total cholesterol, *FPG* fasting plasma glucose, *ALT* alanine aminotransferase, *BUN* blood urea nitrogen, *Cr* serum creatinine concentrations, *WBC* white blood cells count, *hsCRP* high-sensitive CRP, *HOMA*-*IR* homoeostasis model of assessment–insulin resistance

**Fig. 1:**
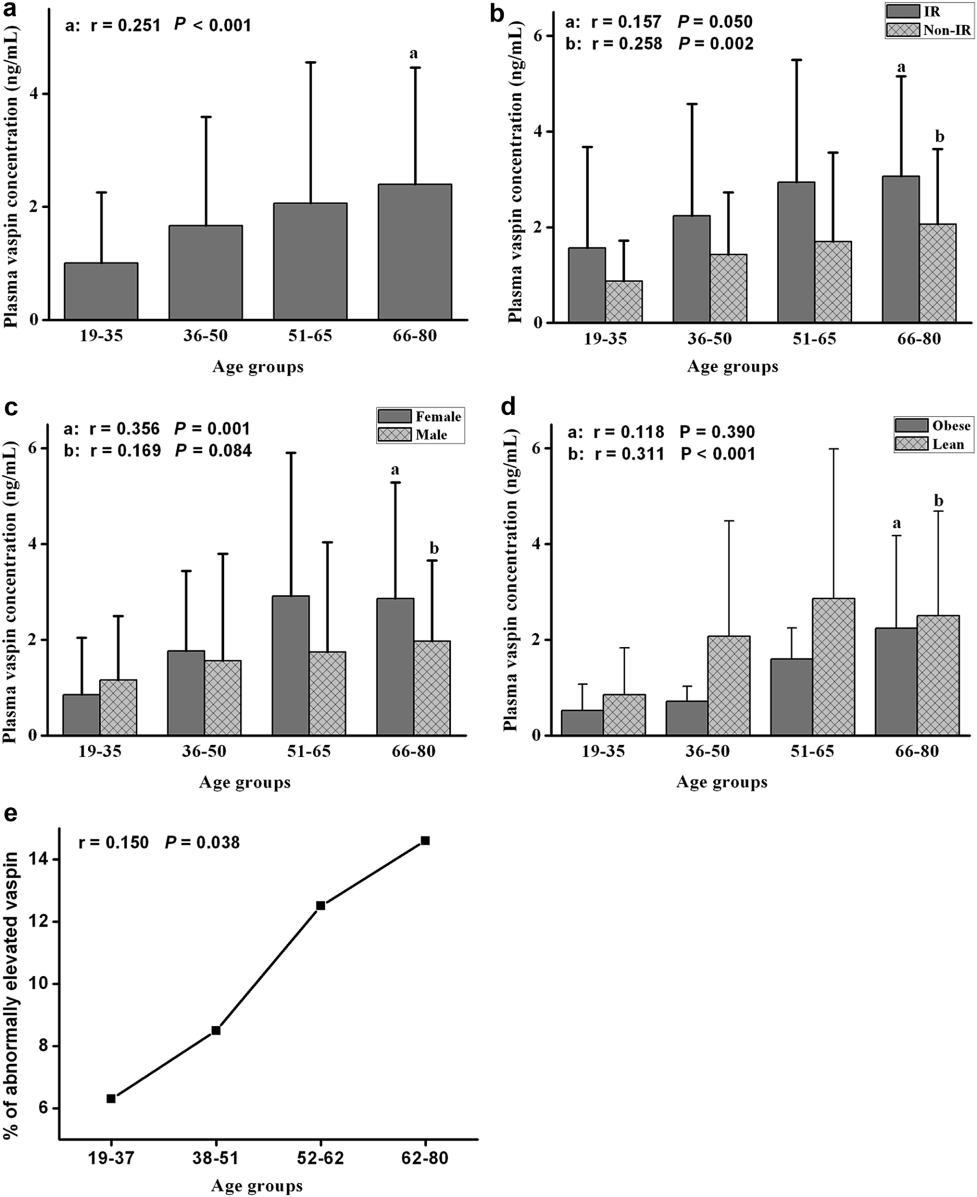
Plasma vaspin concentration in different age groups. **a** Plasma vaspin levels increased with increasing age in whole population. **b** Plasma vaspin concentrations according to age and insulin resistance (IR: HOMA-IR greater than 2.5). **c** Plasma vaspin concentrations increased with increasing age in different sex groups. **d** Plasma vaspin concentrations according to age in obese and lean groups. **e** The trend of increasing prevalence of extremely elevated vaspin with rising age

As expected, in total population analysis, mean circulating vaspin levels were significantly higher in IR group (2.56 ± 3.69 ng/mL) compared non-IR group (1.48 ± 2.62 ng/mL) (*p* < 0.01) (Fig. 1b) and higher in females (2.01 ± 3.10 ng/mL) compared with males (1.61 ± 2.91 ng/mL) (*p* < 0.05) (Fig. 1c).

### Plasma vaspin concentrations increased with age

For all participants combined, plasma vaspin levels increased with ageing (Fig. 1a), from 1.01 ± 2.25 ng/mL for those aged 19–35 years to 1.67 ± 2.95 ng/mL for those aged 36–50 years, 2.05 ± 3.46 ng/mL for those aged 51–65 years and 2.40 ± 3.06 ng/mL for those aged 66–80 years (*r* = 0.251, *p* < 0.001). When divided into subgroups, same trend had been found in both sexes (Fig. 1c), both IR and non-IR subjects (Fig. 1b) and both obese and lean subjects (Fig. 1d). As expected, plasma adiponectin were correlated with increasing age in total (Table 1) or sex-specific analyses (data not show).

### Extremely elevated vaspin ascents with age

Using extremely elevated vaspin cut-off values of 7.66 ng/mL (90 % cut point found in this study), the prevalence of extremely elevated vaspin was lowest in the 19–35 year age group at 6.3 % (3 patients), then subsequently gradually ascents to 8.5 % (4 patients), 12.5 % (6 patients) and 14.6 % (7 patients) for each progressive age subcategory (*r* = 0.150, *p* = 0.038) (Fig. 1e).

### Correlation of vaspin with baseline clinical variables and other biomarkers

In univariate analyses, vaspin plasma level is positively correlated with age (*r* = 0.215, *p* = 0.003), adiponectin, insulin, HOMA-IR and waist–hip ratio in the whole population (Table 2). In HOMA-IR-specific analyses, vaspin plasma level is positively correlated with age (*r* = 0. 285, *p* = 0.002), TC, LDL-C, ALT and HOMA-IR (Fig. 1; Table 2) in non-IR group, while, in sex-specific analyses, vaspin plasma level is positively correlated with age (*r* = 0.356, *p* = 0.001), ALT and adiponectin in women, and is positively related to insulin and HOMA-IR in men (Fig. 1; Table 2). In lean subjects, vaspin plasma level is positively correlated with age (*r* = 0.311, *p* < 0.001), waist–hip ratio, TC, LDL-C, FPG, ALT, adiponectin and HOMA-IR, while in obese subjects, it is correlated with FPG (Fig. 1; Table 2) negatively.

**Table 2:** Correlations between vaspin and other variables in different subgroups

Parameters	Total (*n* = 191)	Insulin resistance		Sex		Obese	
		IR (*n* = 53)	Non-IR (*n* = 138)	Male (*n* = 106)	Female (*n* = 85)	Obese (*n* = 55)	Lean (*n* = 136)
Age	**0.141 (0.052)**	**0.157 (0.050)**	**0.258 (0.002)**	0.169 (0.084)	**0.356 (0.001)**	0.118 (0.390)	**0.311 (<** **0.001)**
Sex	−0.053 (0.464)	0.115 (0.410)	0.092 (0.281)	–	–	−0.068 (0.621)	−0.097 (0.259)
BMI	−0.046 (0.530)	−0.267 (0.054)	0.042 (0.626)	−0.002 (0.987)	−0.002 (0.986)	0.159 (0.247)	0.022 (0.798)
Waist–hip ratio	**0.171 (0.018)**	0.144 (0.304)	0.149 (0.081)	0.157 (0.108)	0.178 (0.103)	0.122 (0.375)	**0.170 (0.048)**
TC	0.044 (0.594)	−0.115 (0.414)	**0.234 (0.006)**	−0.006 (0.948)	0.191 (0.081)	−0.194 (0.156)	**0.279 (0.001)**
Triglycerides	0.015 (0.841)	−0.086 (0.540)	0.126 (0.140)	−0.034 (0.732)	0.117 (0.284)	−0.137 (0.317)	**0.161 (0.061)**
HDL-C	−0.018 (0.806)	0.105 (0.455)	−0.044 (0.606)	0.011 (0.912)	−0.050 (0.650)	−0.149 (0.277)	0.044 (0.609)
LDL-C	**0.156 (0.031)**	−0.050 (0.724)	**0.193 (0.023)**	0.046 (0.643)	0.153 (0.163)	−0.120 (0.381)	**0.233 (0.006)**
FPG	0.104 (0.152)	0.037 (0.795)	0.114 (0.181)	0.072 (0.466)	0.115 (0.294)	−0.254 (0.061)	**0.225 (0.008)**
ALT	0.072 (0.324)	−0.008 (0.953)	**0.261 (0.002)**	0.178 (0.068)	**0.295 (0.006)**	−0.120 (0.383)	**0.322 (0.001)**
BUN	−0.050 (0.494)	0.035 (0.806)	0.042 (0.626)	0.110 (0.261)	0.013 (0.906)	0.213 (0.119)	−0.008 (0.982)
WBC	−0.016 (0.111)	−0.085 (0.546)	−0.154 (0.072)	−0.172 (0.079)	−0.085 (0.440)	−0.127 (0.356)	−0.131 (0.128)
hsCRP	−0.028 (0.842)	0.201 (0.440)	−0.198 (0.254)	−0.0.080 (0.703)	0.293 (0.139)	0.302 (0.196)	0.102 (0.578)
Insulin	0.051 (0.473)	0.151 (0.256)	0.150 (0.079)	**0.171 (0.040)**	0.181 (0.098)	−0.049 (0.722)	0.116 (0.117)
Adiponectin	**0.184 (0.011)**	**0.436 (0.001)**	0.129 (0.131)	0.182 (0.061)	**0.206 (0.049)**	0.207 (0.129)	**0.191 (0.026)**
HOMA-IR	**0.161 (0.026)**	0.144 (0.282)	**0.147 (0.046)**	**0.161 (0.039)**	*0.189 (0.084)*	−0.126 (0.360)	**0.184 (0.032)**

Data were represented as *r* (*p* value)

Bold and italic values indicate *p* values of correlations between vaspin and age in total and in subgroup of insulin resistance, which are no less than 0.05, but they can be considered to have statistical significance as they could be corrected by increased sample size

*BMI* body mass index, *LDL*-*C* low-density lipoprotein cholesterol, *HDL*-*C* high-density lipoprotein cholesterol, *TG* triglycerides, *TC* total cholesterol, *FPG* fasting plasma glucose, *ALT* alanine aminotransferase, *BUN* blood urea nitrogen, *Cr* serum creatinine concentrations, *WBC* white blood cells count, *hsCRP* high-sensitive CRP, *HOMA*-*IR* homoeostasis model of assessment–insulin resistance

### Standard multiple regression analysis of vaspin plasma level

The results of a stepwise multiple regression analysis with vaspin as the dependent variable and age, BMI, waist–hip ratio, insulin, adiponectin, HOMA-IR, FBG, WBC, triglycerides, HDL-C as independent variables are presented in Table 3. Model 1 evaluates the effects of BMI and waist–hip ratio on plasma vaspin levels, which explained 17.1 % of the variance in vaspin levels. In Model 2, indices of glucose metabolism were introduced into the regression analysis which explained an additional 8.6 % on plasma vaspin level. In Model 3, adiponectin, WBC and lipid profile were additionally explained 10.2 % on plasma vaspin level. And in Model 4, age was additionally introduced into the regression analysis to evaluate the combined effect on plasma vaspin concentrations, which altogether explained 43.5 % of the variance in vaspin levels, with age contributing 7.6 % on plasma vaspin level.

**Table 3:** Standard multiple regression analysis for the impact of age and other potential confounders on vaspin levels

Independent variables	Non-standardized *β*-coefficients	95 % CI	*p* value
Model 1 (*R* ^2^ = 0.171, *F* = 2.844, *p* = 0.061)			
BMI	0.062	0.016 to 0.108	0.008
Waist–hip ratio	1.790	0.468 to 3.112	0.008
Model 2 (*R* ^2^ = 0.257, *F* = 2.612, *p* = 0.026)			
Model 1 plus			
Insulin	−0.087	−0.220 to 0.046	0.200
FBG	−0.163	−0.438 to 0.112	0.245
HOMA-IR	0.453	−0.148 to 1.053	0.139
Model 3 (*R* ^2^ = 0.359, *F* = 2.402, *p* = 0.008)			
Model 2 plus			
Adiponectin	0.231	−0.093 to 0.555	0.161
TG	0.132	−0.026 to 0.291	0.101
LDL-C	0.171	−0.100 to 0.442	0.215
WBC	−0.084	−0.149 to −0.019	0.012
Model 4 (*R* ^2^ = 0.435, *F* = 3.454, *p* < 0.001)			
Model 3 plus			
Age	0.174	0.080 to 0.269	<0.001

*BMI* body mass index, *FPG* fasting plasma glucose, *HOMA*-*IR* homoeostasis model of assessment–insulin resistance, *LDL*-*C* low-density lipoprotein cholesterol, *WBC* white blood cells

## Discussion

In this population-based, wide age-ranged, normal group of Chinese people, plasma vaspin concentration increased with ageing for both sexes, both IR and non-IR subjects and both obese and lean subjects. The correlation between rising age and increasing vaspin remained significant even after multivariate adjustments for factors such as sex, BMI, waist–hip ratio, indices of glucose metabolism, lipid profile and adiponectin. The prevalence of extremely elevated vaspin was also increased with increasing age quartiles. These results suggest that the age-related increase in plasma vaspin levels is a common phenomenon in normal subjects and that age was an independent predictor of plasma vaspin level.

Ageing is related to fat redistribution, which is characterized by loss of peripheral subcutaneous fat and accumulation of central fat. Age-related fat loss or lipoatrophy is a major factor for adipose tissue dysfunction in centenarians [7]. Moreover, adipokine dysregulation is paradoxically correlated with lipodystrophy and lipoatrophy with ageing [6]. Increments in fat mass especially visceral fat and hyperinsulinemia may modulate vaspin expression and release [8, 9, 12]. In analysis of our population characteristics, BMI, waist–hip ratio and adiponectin increased with age, suggesting that central obesity increased with age. Since plasma vaspin significantly correlated with age, waist–hip ratio, fasting plasma insulin concentration and HOMA-IR, it is conceivable that with ageing, which is typically associated with alterations in body fat distribution (increased proportion of visceral fat) and insulin resistance, vaspin expression would be up-regulated.

In addition, vaspin levels were found to be significantly correlated with some markers of lipid metabolism such as TC, TG and LDL-C, which indicates that vaspin may be induced by dyslipidemia as a compensatory mechanism, especially because vaspin is an adipokine secreted by adipocytes. The latest literatures reported that vaspin could activate cell surface GRP78 complex [30]. And GRP78 expression and activity are declined with ageing [31]. So another possible interpretation for the age-related increase in plasma vaspin levels is positive feedback due to down-regulation or resistance of vaspin receptors with age, but we could not measure vaspin receptors in this study to investigate this possibility. It has been reported that vaspin levels are negatively correlated with serum follicle-stimulating hormone levels, sex hormone-binding globulin levels and positively correlated with free androgen index in women with polycystic ovary syndrome [27]. Our present study found that plasma vaspin increased in both males and females but to a smaller extent in males. The sexual dimorphism in the levels of circulating vaspin also had been found previous study [12, 21, 26]. Therefore, another plausible explanation of the age-related increase in plasma vaspin levels is a decrease in sex hormones with age. Our finding that plasma vaspin levels were significantly increased in subjects >50 years old supported this hypothesis.

Ageing is often accompanied by reduced insulin sensitivity. A relationship between vaspin with insulin and HOMA-IR was observed in the current study. Previous study also reported that circulating vaspin levels is significantly correlated with insulin sensitivity in subjects with normal glucose tolerance [12] and in obese children [8], suggesting a defensive role of vaspin against insulin resistance. The reported insulin-sensitizing effect of vaspin on adipose tissue supports this possibility [10]. Previous studies reported that vaspin suppressed the expression of several inflammatory factors, such as tumour necrosis factor (TNF)-α, leptin and resistin [10, 16]. It also attenuated TNF-α-induced p65 phosphorylation, reduces intercellular adhesion molecule-1 expression and reactive oxygen species production, inhibits vascular smooth muscle cells proliferation and chemokinesis [17–19], increased nitric oxide bioavailability in vascular endothelial cells [32], and protected vascular endothelial cells against free fatty acid-induced apoptosis. Thus, high plasma vaspin levels in the elderly might have numerous beneficial effects because inflammation, atherosclerosis and apoptosis are advanced with age. Vaspin was positively associated with adiponectin. Higher adiponectin levels are related to the longevity. However, it is still unclear whether higher plasma levels also related to the longevity.

The strengths of this study include the use of a population-based, wide age-ranged, well-characterized group of normal people. Some limitations should be considered in this study. Firstly, this was a cross-sectional rather than a longitudinal study. The cross-sectional design made it difficult to determine the causality of observed relationships. In addition, we did not assess habitual physical activity and menopausal status, which may affect vaspin and adiponectin levels.

In conclusion, vaspin level was not constant, but increased with increasing age, independent of sex, indices of glucose metabolism, surrogate markers of adiposity, lipid profile and other markers of adiposity such as adiponectin. On the basis of these results, vaspin might be used as a predictor of cardiovascular disease in the future; we consider that clinicians should take into account age-related changes in plasma vaspin levels when interpreting a patient’s plasma vaspin level in clinical practice.

## Abbreviations

TNF-α: Tumour necrosis factor-α
BMI: Body mass index
HOMA-IR: Homoeostasis model of assessment–insulin resistance
IR: Insulin resistance
WBC: White blood cell count
hsCRP: High-sensitive C-reactive protein
FPG: Fasting plasma glucose
ALT: Alanine aminotransferase
BUN: Blood urea nitrogen
TC: Total cholesterol
LDL-C: Low-density lipoprotein cholesterol
HDL-C: High-density lipoprotein cholesterol
TG: Triglycerides
EGTA: Ethylene diamine tetraacetic acid
